# The basal ganglia mediate the inter-hemispheric transfer of complex tool-use skill

**DOI:** 10.1016/j.isci.2025.114523

**Published:** 2025-12-23

**Authors:** Sayori Takeda, Kouji Takano, Kimihiro Nakamura

**Affiliations:** 1 Section of Systems Neuroscience, National Rehabilitation Center for Persons with Disabilities, Tokorozawa 359-8555, Japan

**Keywords:** Behavioral neuroscience, Cellular neuroscience

## Abstract

Motor skills learned in one hand generalize to the other hand via plastic changes in motor systems. Such “intermanual transfer” may arise during complex tool-use learning, but its neural underpinnings remain unknown. Using resting-state fMRI, we explored neurobehavioral effects occurring while right-handed participants were trained to use a novel complex tool with their left hand. Behaviorally, training improved tool-use performance equally for both hands, demonstrating a robust effect of intermanual transfer. For both hands, this behavioral effect correlated with functional connectivity changes between right dorsal premotor cortex (PMd) and intraparietal sulcus (IPS). For the untrained right hand, additional change emerged in right basal ganglia (BG), which showed increased behavior-connectivity correlation with bilateral PMd. Thus, tool-use skill learned by left-hand training is represented in the right PMd-IPS network and transferred to left PMd responsible for right-hand performance, pointing to a pivotal role of BG in generalizing complex tool-use skill across hands.

## Highlights

• Complex tool-use skill learned in the left hand generalizes to the right hand
• The right PMd-IPS network is established during left-hand training
• The right BG-left PMd connectivity reflects untrained right-hand performance
• The BG mediate the rapid intermanual transfer of complex tool-use skill

## Graphical abstract

**Figure:**
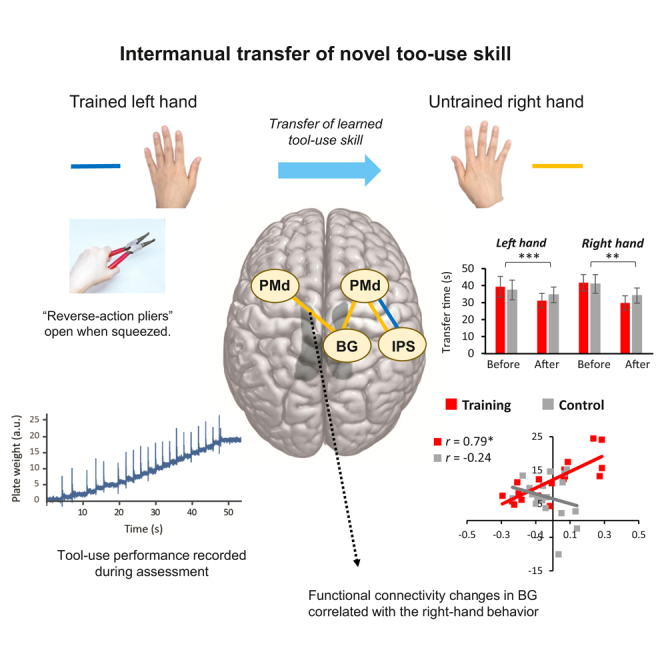

## Introduction

Manipulating tools such as scissors and pliers is a learned psychomotor skill closely linked to our daily life and partially relies on neurobiological mechanisms shared by non-human primates.^1^ However, unlike simple motor actions such as tapping and grasping, human tool-use is characterized by its heavy reliance on multiple cognitive components beyond the motor domain, including semantic knowledge, spatial transformation, and body schema.^2^^,^^3^ It is therefore likely that motor skill knowledge of human tool-use involves broader and higher order brain regions outside the neural system involved in motor execution.

For both humans and primates, a well-known general property of motor skills is that a specific skill learned with one hand generalizes to the other hand.^4^^,^^5^ Such “intermanual transfer” is also shown to rapidly occur for human tool-use skills even after a brief practice,^6^^,^^7^^,^^8^ but little is known about neural mechanisms responsible for this complex behavioral phenomenon. Namely, although the intermanual transfer of simple motor skills (e.g., sequential tapping) has been associated with several distinct brain regions, including the supplementary motor area (SMA),^5^^,^^9^ dorsal premotor cortex (PMd),^10^ and posterior parietal lobule,^11^ it remains open whether the same neural systems are involved in the rapid generalization of more complex tool-use skills across hands. Rather, given that human tool-use involves multiple cognitive components, its intermanual transfer may rely on different neural systems interacting with a broader tool-use network.

On the theoretical side, three different neurocognitive models have been proposed to account for the intermanual memory transfer in motor learning. First, the “cross-activation model” posits that unilateral motor learning concurrently activates homologous motor systems in the left and right hemispheres. According to this model, motor skill memory (engram) is established in both the contralateral hemisphere responsible for the trained hand and the ipsilateral hemisphere responsible for the untrained hand, thereby improving motor performance for the untrained hand.^12^^,^^13^^,^^14^ Second, the “callosal access model” assumes that motor memory is acquired in the dominant left hemisphere regardless of which hand is trained.^15^ Motor engrams are thus thought to be stored in the left hemisphere even after left-hand training, which can be accessed from the right hemisphere motor systems via the corpus callosum. Third, the “bilateral access model” proposes that motor engrams shaped by unilateral training are encoded as abstract representations independent of specific effectors (i.e., the trained hand) and stored in brain regions accessible by both hands, enabling the untrained hand to benefit from acquired motor skills.^14^^,^^16^^,^^17^

**Figure 1:**
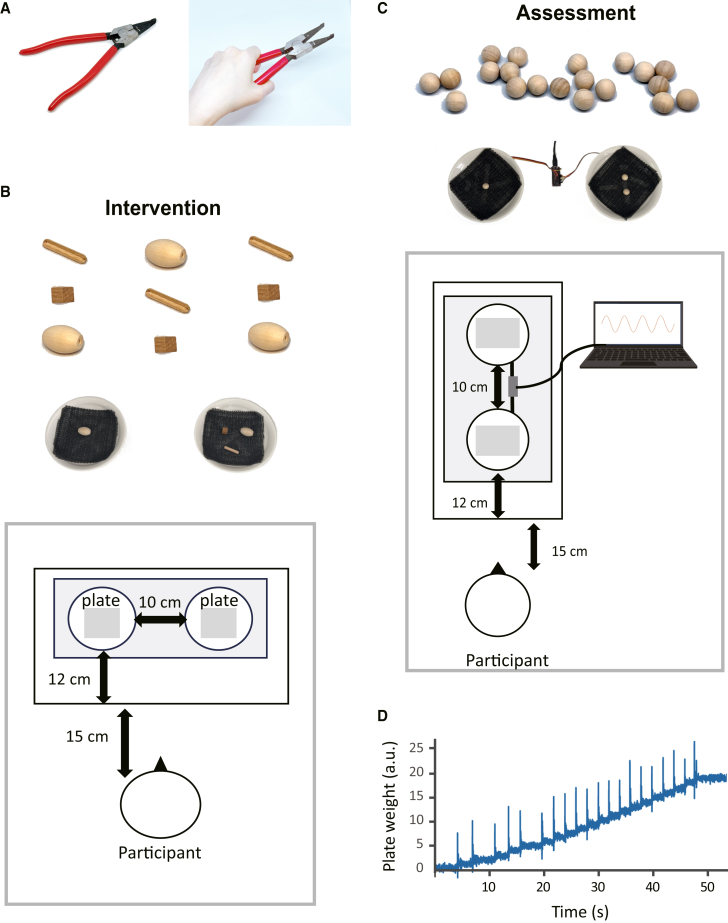
Behavioral tasks (A) A special pair of “reverse-action pliers,” which open when squeezed, was used for intervention and assessment. (B) For intervention, a tool-use training task was performed using three small wooden blocks of three different shapes and two round plastic plates placed side by side. Participants in the training group used the novel tool to transfer these blocks one-by-one from a plate to another, whereas those in the control group simply held the same pliers in their left hand without moving them. (C) Twenty wooden balls were used for the assessment task before and after intervention. Unlike in the training task, the two plastic plates were placed in the front-to-back direction and connected to a laptop PC via a custom-built device to precisely detect and record temporal changes in plate weight. Participants in the training and control groups transferred those balls one by one from the front plate to the back plate using the reverse-action pliers in their left or right hand. (D) Temporal evolution of plate weight during the assessment task in a representative participant.

**Figure 2:**
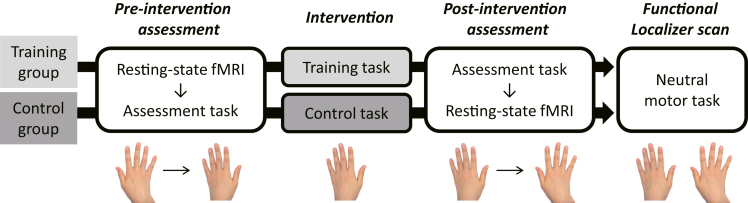
Experiment procedures All participants first underwent resting-state fMRI scans, followed by a pre-intervention assessment. They performed the assessment task first with their right hand and then with the left hand in the fixed order. During intervention (∼16 min), participants in the training and control groups performed the tool-use training task and control task, respectively. All participants then underwent a post-intervention assessment followed by rsfMRI scans. Here, they performed the assessment task first with their left hand and then with the right hand (i.e., in the reverse order of the pre-intervention assessment). Finally, all participants received functional localizer scans in a task-based fMRI experiment in which they manipulated chopsticks with their left or right hand.

In the present study, we addressed the issue using a unique tool-learning paradigm with resting-state fMRI (rsfMRI). Specifically, we explored neural mechanisms involved in the rapid intermanual transfer of tool-use skill, which may occur when right-handed participants learn to use a novel tool with their non-dominant left hand (Figures 1 and 2). Since resting-state functional connectivity (RSFC) has been widely used to identify neural plasticity associated with behavioral improvement in motor skills,^18^ we examined learning-induced changes in RSFC within the neural network for tool-use. Given the well-known role of the dorsal premotor hand area in object manipulation,^9^^,^^14^ the cross-activation model would predict that after training, the left and right PMd equally increase functional connectivity with other regions in the tool-use network. According to the “callosal access model,” however, RSFC changes associated with tool-use learning should emerge only in the dominant left hemisphere even after left-hand training. Lastly, the bilateral access model would predict some other neural systems linking the left PMd responsible for the untrained right hand with the right-hemisphere motor systems responsible for the trained left hand.

## Results

### Behavioral performance

**Figure 3:**
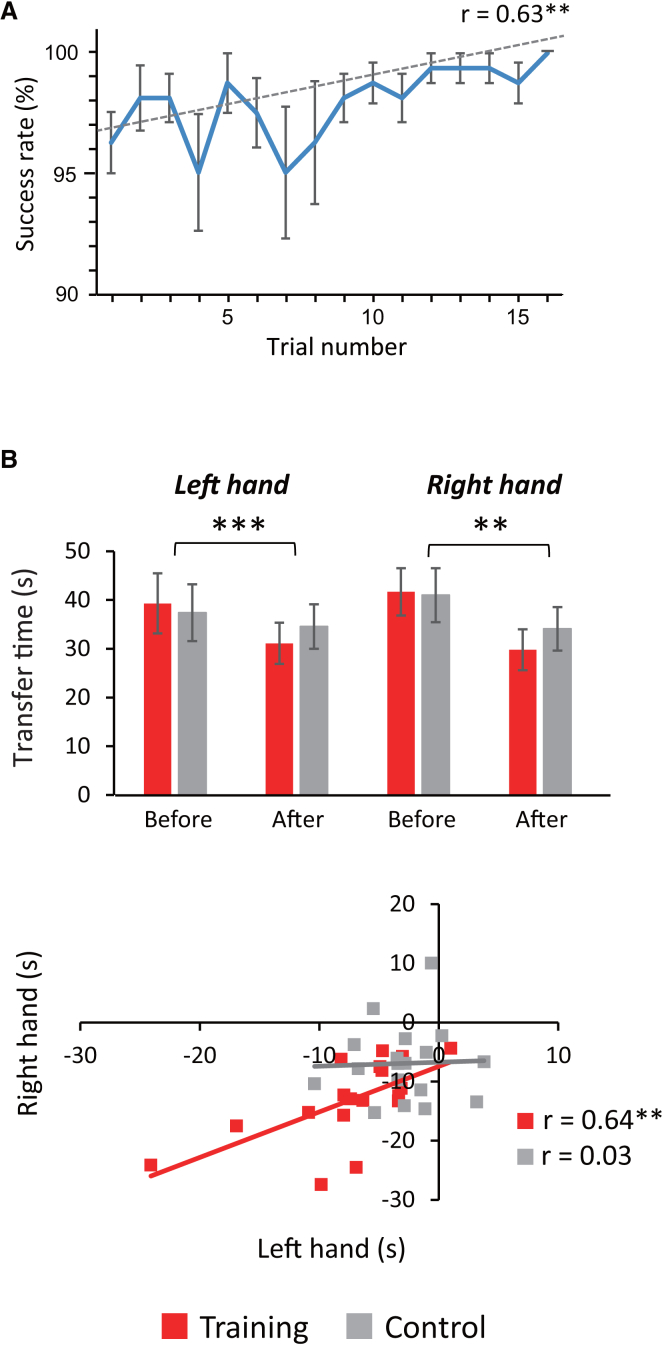
Behavioral performance (A) Temporal changes in tool-use performance during intervention in the training group. Across-participants mean (SEM) rates of successfully transferred objects increased over 16 trials during the training task (r = 0.63, *p* = 0.009). (B) Training-induced changes in transfer time during assessment. Top, significant interaction between intervention (before vs. after) and group (training vs. control) was observed not only for the trained left hand but also for the untrained right hand (mean ± SEM). Asterisks in this and subsequent figures indicate significant interaction between Intervention and Group at ∗∗*p* < 0.01 and ∗∗∗*p* < 0.001, respectively (Table S1). Bottom, behavioral improvements in the right hand plotted against those in the left hand. Behavioral improvements for each hand were obtained by calculating the magnitude of reduction in transfer time after intervention. Significant correlation between the left- and right-hand performance was observed for the training group but not for the control group (see results).

For the training group, temporal changes in tool-use performance during intervention are illustrated in Figure 3A. As expected, behavioral performance progressively improved with trials during the training task (r = 0.63, *p* = 0.009). Next, mean transfer times during the pre- and post-intervention assessments are presented for each hand for each group in Figure 3B. A preliminary analysis with Shapiro-Wilk test confirmed a normal distribution of the transfer time data (*p* > 0.05). To examine behavioral changes in tool-use performance after training, these transfer time data were log-transformed and submitted to a 2 × 2 × 2 repeated measures ANOVA with the effects of Hand (trained left vs. untrained right) and Intervention (before vs. after) as within-participant factors and the effect of Group (training vs. control) as a between-participants factor.

The main effect of Intervention was highly significant (F [1, 34] = 99.60, partial eta squared [η^2^_p_] = 0. 746, *p* < 0.001), whereas those of Hand and Group were both non-significant (F [1, 34] = 1.82, η^2^_*p*_ = 0.051, *p* = 0.187 and F [1, 34] = 1.76, η^2^_*p*_ = 0.049, *p* = 0.193, respectively). Significant interaction was observed between Hand and Intervention (F [1, 34] = 17.85, η^2^_*p*_ = 0.344, *p* < 0.001), suggesting greater effects of Intervention for the untrained right hand than for the trained left hand (9.4 s vs. 5.3 s). Interaction was also significant between Intervention and Group (F (1, 34) = 13.21, η^2^_*p*_ = 0.280, *p* < 0.001), suggesting greater effects of Intervention for the training group than for the control group (9.8 s vs. 4.9 s). No significant interaction was found between Hand and Group (F (1, 34) = 0.39, η^2^_*p*_ = 0.011, *p* = 0.537). Importantly, a three-way interaction was non-significant between Hand, Intervention, and Group (F [1, 34] = 0.07, η^2^_*p*_ = 0.002, *p* = 0.800), suggesting that net effects of intervention (i.e., differences in transfer time between the training and control groups) did not differ in magnitude between the left and right hands (5.3 s vs. 5.0 s).

Since our main interest was to validate the inter-manual transfer of complex tool-use skill, we additionally looked at the impact of intervention on the behavioral performance for the untrained hand. That is, when the analysis was restricted to the right hand, the effect of Intervention was highly significant (F [1, 34] = 84.38, η^2^_*p*_ = 0.713, *p* < 0.001), whereas that of Group was non-significant (F [1, 34] = 2.74, η^2^_*p*_ = 0.075, *p* = 0.107). Critically, there was a significant interaction between Intervention and Group (F [1, 34] = 7.48, η^2^_*p*_ = 0.180, *p* < 0.01), again suggesting greater effects of Intervention for the training group than for the control group (12 s vs. 7 s) (see Table S1 for further analysis).

To visualize the overall effects of learning for each group, individual-level reductions in transfer time for the untrained right hand were plotted against those for the trained left hand (Figure 3B). This analysis revealed significant correlation between the left- and right-hand performance for the training group (r = 0.64, Z = 0.75, and *p* = 0.004) but not for the control group (r = 0.04, Z = 0.04, and *p* = 0.880). This difference in correlation strength was significant between the two groups (Z = 1.97 and *p* = 0.048), suggesting that intermanual transfer was greater in magnitude for the training group than for the control group. Collectively, these findings demonstrate robust effects of intermanual transfer of tool-use skill, whereby brief training in the non-dominant hand facilitated behavioral performance in the untrained dominant hand.

In addition, we performed two supplemental analyses to more closely examine the effects of Hand and Intervention on behavioral performance. First, while we initially expected that our right-handed participants should perform better with their dominant hand than with their non-dominant hand, we found that they overall performed the assessment task faster by 3.2 s with the left hand than with their right hand even before intervention (Figure 3B). Indeed, when the analysis was restricted to the pre-intervention assessment, this difference between hands in transfer time was significant (F [1, 34] = 10.89, η^2^_*p*_ = 0.243, *p* = 0.002). Although rather unexpected, this finding can be interpreted as suggesting that motor skill memory for the typical use of common pliers is much more robust in the dominant hand than in the non-dominant hand, which interfered with the initial use of novel reverse-action pliers more greatly in the right hand than in the left hand.

Second, we also found that participants in the control group performed the assessment task faster by 4.9 s after intervention even though they merely held the tool in their left hand during intervention. When the analysis was restricted to the control group, this effect of intervention on transfer time was significant (F [1, 17] = 32.75, η^2^_*p*_ = 0.658, *p* < 0.001), with its magnitude being greater for the right hand than for the left hand (6.9 s vs. 2.8 s, F [1, 17] = 5.76, η^2^_*p*_ = 0.253, *p* = 0.028). This finding can be interpreted as suggesting that participants in the control group learned to use the novel tool during the pre-intervention assessment, rather than during intervention (see Table S1 for further analysis).

### RSFC associated with intermanual transfer

**Figure 4:**
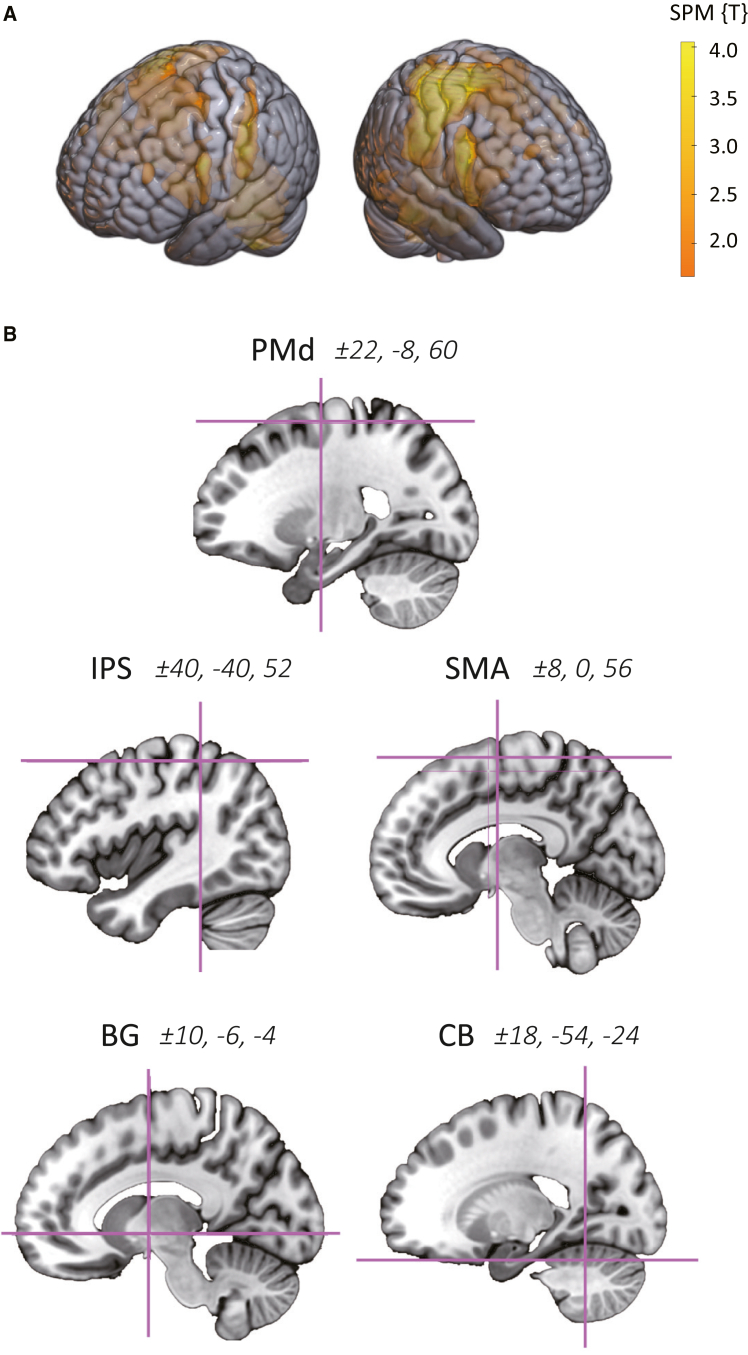
Regions-of-interest used for RSFC analysis (A) Brain regions involved in tool-use identified by the whole-brain analysis of functional localizer scans. (B) The resulting statistical parametric map was used to create five regions-of-interest in each hemisphere by reference to the known Montreal Neurological Institute (MNI) co-ordinates for each region (see results).

We first performed an independent functional localizer scan to isolate brain regions involved in tool-use, which yielded five regions-of-interest (ROIs), including the PMd, intraparietal sulcus (IPS), SMA, basal ganglia (BG) and cerebellum (CB) in each hemisphere (Figure 4). For each of the left and right PMd, we then calculated learning-related changes in RSFC with other ROIs and examined how these changes in RSFC correlated with behavior (see method details in STAR Methods).

**Figure 5:**
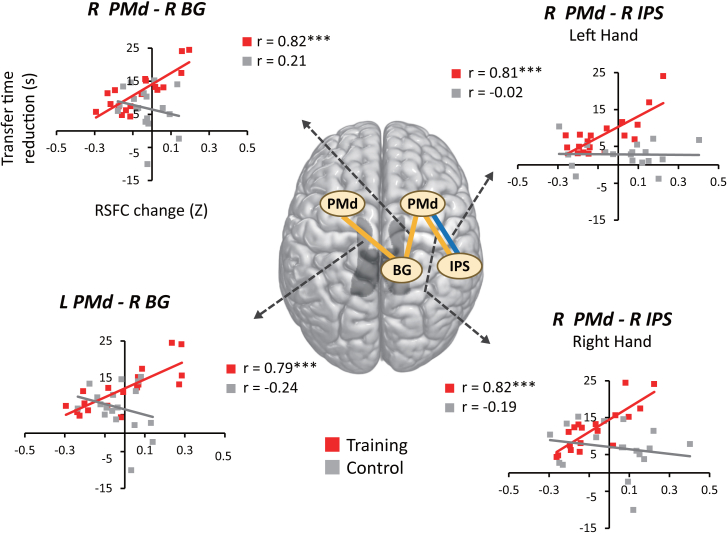
RSFC changes associated with behavioral improvements Blue and yellow lines indicate neural connections where changes in RSFC correlated with behavioral improvement in the left and right hands, respectively. These correlations were all significantly greater in strength for the training group than for the control group (see results). In particular, only the right BG showed significant behavior-RSFC correlation with the left PMd responsible for the untrained right hand.

Correlations between the behavioral effects of intervention and changes in RSFC with PMd seeds are summarized in Table 1. For the trained left hand, while no brain region emerged as significant in factorial whole-brain analysis (see method details in STAR Methods), this parametric analysis identified the PMd-IPS connection in the right hemisphere (Figure 5), which showed significant correlation with behavior in the training group (*Z* = 1.13, *p* = 0.001) but not in the control group (*Z* = −0.02, *p* = 0.941). Indeed, this between-group difference in correlation strength was significant (*p* = 0.018).

**Table 1:** Correlations between behavioral improvements and functional connectivity with the PMd

Hand	Seed	ROI	Training		Control		Training vs. control	
			*Z*	*p*	*Z*	*p*	*Z*	*p*
Left	R PMd	L PMd	−0.14	0.884	0.19	0.815	0.90	0.659
		L SMA	0.04	0.891	0.18	0.704	0.38	0.901
		R SMA	0.07	1.000	0.57	0.243	1.36	0.770
		L IPS	0.17	0.900	−0.28	1.170	1.23	0.654
		R IPS	1.13	<**0.001**	−0.02	0.941	3.15	**0.018**
		L BG	0.05	0.956	0.26	0.906	0.84	0.594
		R BG	0.51	0.221	0.16	0.674	0.96	0.761
		L CB	0.22	1.155	0.20	0.887	0.03	0.978
		R CB	0.19	1.024	0.07	0.878	0.30	0.858
	L PMd	R PMd	−0.14	1.752	0.19	0.582	0.90	1.098
		L SMA	−0.14	1.325	0.24	0.599	1.04	1.341
		R SMA	−0.15	2.507	0.13	0.681	0.77	0.797
		L IPS	−0.03	1.026	0.20	0.635	0.63	0.794
		R IPS	0.06	1.057	0.47	0.585	1.12	2.358
		L BG	−0.02	0.942	0.27	0.531	0.79	0.961
		R BG	0.35	1.530	0.30	0.687	0.14	0.891
		L CB	−0.14	1.066	−0.32	0.922	0.52	0.775
		R CB	−0.11	0.999	0.05	0.846	0.44	0.744
Right	R PMd	L PMd	−0.21	0.617	−0.08	0.838	−0.36	1.074
		L SMA	−0.06	0.816	−0.15	0.828	0.25	0.903
		R SMA	0.20	0.540	−0.47	0.630	1.85	0.195
		L IPS	0.26	0.536	0.10	0.905	0.45	1.462
		R IPS	1.15	<**0.001**	−0.19	0.819	3.69	**0.002**
		L BG	0.41	0.309	0.37	0.626	0.13	0.898
		R BG	1.15	<**0.001**	−0.21	1.194	3.75	**0.001**
		L CB	−0.11	0.749	0.04	0.868	−0.41	1.224
		R CB	0.32	0.475	0.21	0.905	0.29	0.988
	L PMd	R PMd	−0.20	0.739	−0.08	0.745	−0.33	0.829
		L SMA	0.29	1.152	−0.24	0.756	1.46	0.324
		R SMA	0.26	0.909	−0.38	1.197	1.79	0.328
		L IPS	−0.26	0.689	0.32	0.918	−1.58	0.345
		R IPS	−0.11	1.002	0.14	0.672	−0.66	0.916
		L BG	−0.07	1.000	0.14	0.754	−0.61	0.818
		R BG	1.07	<**0.001**	−0.24	1.005	3.60	**0.003**
		L CB	−0.05	0.948	−0.16	0.770	0.33	0.740
		R CB	0.05	0.853	0.21	0.688	−0.47	0.336

Behavioral improvements for each hand were calculated as the magnitude of reduction in transfer time after intervention. Z and *p* values represent Fisher-transformed correlation coefficients and FDR-corrected *p* values, respectively.

For the untrained right hand, we again found significant correlation between behavior and RSFC change in the PMd-IPS connection in the right hemisphere (*Z* = 1.15, *p* < 0.001). As shown in Figure 5, we found two additional neural connections associated with behavioral improvement in the training group, i.e., between the right PMd and right BG (*Z* = 1.15, *p* < 0.001) and between the left PMd and right BG (*Z* = 1.07, *p* < 0.001). These correlations were all significantly greater in strength than those observed for the control group, which showed no significant correlation between behavior and RSFC (Table 1). These effects of intervention observed in the right-hemisphere are clearly inconsistent with the callosal access model positing that learned motor memory is stored in the dominant left hemisphere regardless of which hand is trained. They are also at odds with the bilateral activation model according to which motor engrams shaped by unilateral motor learning are stored in homologous areas in the left and right hemispheres.

**Figure 6:**
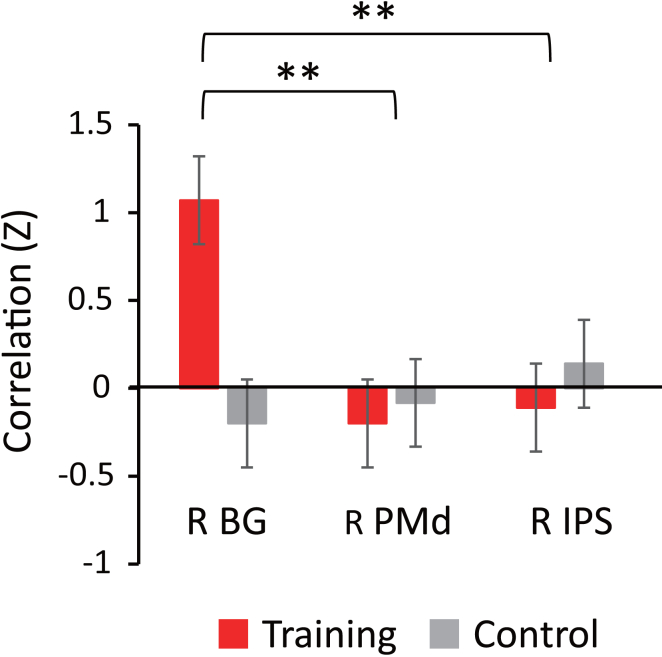
RSFC-behavior correlation strength in functional connectivity of the left PMd Since significant correlation with the untrained right-hand performance was found in the right PMd, IPS, and BG (Figure 5), their correlation strengths with the left PMd responsible for the same hand (mean ± SEM) were directly compared between the three sites. Pairwise comparisons confirmed that correlation strength with behavior was greater for the left PMd-right BG connection than for the left PMd-right PMd and left PMd-right IPS connections (see results), suggesting that the functional connectivity with the right BG contributes more greatly to the observed behavioral improvement as compared to the other two regions in the right hemispheres.

In addition, since three right-hemisphere regions (i.e., PMd, IPS, and BG) were identified as showing significant RSFC-behavior correlations with the untrained right-hand performance (Table 1), we further examined whether the right BG specifically contributes to interhemispheric transfer by comparing its correlation strength with the left PMd responsible for the right hand (Figure 6). That is, although only the BG showed significant correlation with the left PMd, we asked whether the correlation strength with the left PMd was substantially greater for the right BG than for the right PMd and IPS. To this end, we compared these correlation measures by treating Site (PMd, IPS, and BG) as a within-participant factor and Group (training and control) as a between-participant factor (see method details in STAR Methods). While the effects of Site and Group were both non-significant (*p*_within_ = 0.075 and *p*_between_ = 0.152), we found significant evidence that RSFC-behavior correlation with the left PMd differed in strength between the three sites (*p*_intearction_ < 0.001). Pairwise comparisons confirmed that behavior-RSFC correlation strength with the left PMd was significantly greater for the right BG as compared to the right PMd and IPS (false discovery rate [FDR]-corrected *p*_intearction_ = 0.009 and *p*_intearction_ = 0.002, respectively), whereas it did not differ between the right PMd and right IPS (*p*_intearction_ > 0.5). Taken together, these findings suggest that the functional connectivity between the left PMd and right BG specifically contributes to the behavioral improvement observed in the untrained right hand.

## Discussion

Using rsfMRI, we explored neurobehavioral mechanisms involved in the intermanual transfer of complex tool-use skill. To this end, we employed a highly atypical type of pliers which open when squeezed (Figure 1). This special tool can be considered sufficiently novel, because none of our participants had known or used similar tools in the past. Its use also proved to be complex enough for participants, because they performed more slowly with the dominant right hand than the non-dominant left hand during the pre-intervention assessment.

Our behavioral results demonstrate a rapid intermanual transfer of tool-use skill, in which brief training of the non-dominant hand (∼16 min) improves tool-use performance in the untrained right hand. This finding well concurs with some behavioral studies showing effective intermanual transfer after a brief practice with similar time frames.^6^^,^^7^^,^^8^ Importantly, although our supplemental analyses revealed that participants in the control group also learned to use the novel tool during the initial assessment, we demonstrated substantial effects of the intervention—independent of such incidental learning during assessment—by showing a significant interaction between Intervention and Group at both the behavioral and neural levels. Given that all reported between-group differences were obtained by testing the Intervention × Group interactions, they can be explained only as arising from the stage of Intervention, rather than from the stage of assessment.

The present results are overall consistent with the view that motor skill acquisition occurs rapidly during the initial stage of motor learning.^19^^,^^20^ While this process may depend on the type and difficulty of tasks,^19^^,^^20^ the present results suggest that a complex skill for novel tool-use can be effectively acquired in the trained hand even with short practice time when task difficulty is appropriately controlled. The observed effects of tool-use learning can be partially attributed to cognitive components outside the motor domain, because our object manipulation task required cognitive-level adjustments to modify the prior motor knowledge about the use of ordinary pliers. While behavioral improvements in the trained left hand strongly correlated with those in the untrained right hand, this finding is well consistent with the previous work by Perez et al.^5^^,^^21^ showing that the degree of memory transfer to the untrained hand is influenced by the magnitude of skill acquisition in the trained hand during finger movement tasks.

At the neural level, we found that behavioral improvement in the trained left-hand correlated with the PMd-IPS connection within the right hemisphere. Since such changes in RSFC following motor learning generally reflect offline consolidation of motor memory acquired through training,^22^ this finding likely captures neural traces of learning, rather than transient neural effects associated with motor execution, and well concurs with the fact that the IPS is involved in several sensorimotor components associated with tool-use, such as bodily movement, integration of visual and somatosensory information,^23^^,^^24^^,^^25^^,^^26^ grasping and manipulation of objects^27^ and online control of movement.^28^ The observed positive correlation between behavior and RSFC thus suggest that the relative contribution of the IPS increases with the progression of motor learning in the PMd.

For the untrained right-hand, we found that behavioral improvements correlated with RSFC in the same right PMd-IPS connection and two additional neural connections linking the right BG with the left and right PMd. These RSFC changes associated with right-hand performance are thus likely to reflect the inter-hemispheric transfer of tool-use skill acquired in the trained left hand. On the one hand, the right PMd-IPS connection involved in the trained left hand was again correlated with behavioral improvement in the untrained right hand. While both of these brain regions have been associated with the encoding of learned movements and tool-use skills,^25^^,^^27^^,^^28^ this finding is particularly in good accordance with previous studies showing that the PMd and posterior parietal area are involved in the intermanual transfer of motor skills in both humans and primates.^10^^,^^29^ In fact, such encoding of motor memory is likely to generalize more broadly, occurring not only between the left and right hands but also across different effectors, e.g., between the eyes and hands.^27^^,^^30^

We also found that the untrained right-hand performance correlated with intra-hemispheric and inter-hemispheric connections linking the right BG with the bilateral PMd. While the BG are generally involved in the learning, planning, and execution of novel motor skills via their neural connections with multiple cortical areas, including the PMd,^31^^,^^32^ the present finding well concurs with the notion that the BG constitute a part of the tool-use network^33^^,^^34^ by mediating the encoding and execution of specific motor programs.^14^^,^^35^ Notably, BG are thought to play a key role in the temporal or hierarchical “sequencing” of such neurocognitive components required for motor behavior,^36^^,^^37^ which seems critically important for tool-use skills as well. Accordingly, while intermanual transfer during sequential motor tasks has been associated with restricted brain regions, such as the SMA, subcortical nuclei,^4^^,^^5^ and PMd,^10^ our results suggest that the right BG contribute to the hemispheric transfer of more complex tool-use skill by sequencing the broader right-hemisphere tool-use systems responsible for the trained left hand.

By comparing the behavior-RSFC correlation strength at the left PMd, we further confirmed that the right BG contributed more greatly to the behavioral improvement in right-hand performance than the right PMd and IPS (Figure 6). Although the left and right premotor areas are thought to be strongly connected to each other, both structurally and functionally,^14^ this finding suggests that the interhemispheric connections in PMd are not responsible for the observed improvement in right-hand performance and thus are inconsistent with the callosal access model whereby motor engrams stored in the left hemisphere are accessed from the right hemisphere motor systems via the corpus callosum.^15^ Rather, our results are more consistent with the bilateral access model assuming that motor skill memory formed through unilateral training is stored as abstract representations in brain regions accessible by both the trained and untrained hands.^14^^,^^16^^,^^17^

To summarize, the present results show that (1) the neural network between the PMd and IPS is rapidly established in the right hemisphere during left-hand training for novel tool-use and (2) this motor skill memory can facilitate the untrained right-hand performance via the functional connectivity between the right BG and the left PMd. While previous research has identified neural structures involved in the intermanual transfer of simple motor memory, our results extend the existing knowledge by showing that such intermanual transfer occurs effectively for more complex motor skill of tool-use, whereby the BG play a pivotal role in mediating the inter-hemispheric transfer between the left and right motor systems. Given that difficulty in the use of everyday tools is a highly common sequela of stroke and other neurological disorders, our results have practical implications for neurorehabilitation by showing that training the non-dominant hand has the potential to facilitate functional recovery of tool-use skills in the paralyzed dominant hand. While conventional rehabilitation training typically focuses on the paretic hand and avoids intensive use of the non-paretic hand to prevent possible interhemispheric inhibitions,^38^ the present results broaden our understanding of the neural mechanisms underlying the rapid intermanual transfer in tool-use, warranting further investigation for clinical application as an alternative rehabilitation method.

### Limitations of the study

It should be noted as limitations of the study that the sample size might be relatively small, meaning that the findings reported in the study require further confirmatory investigations with larger samples. Moreover, the pre- and post-intervention assessments in the present study focused only on the short-term effects arising from low-complexity tool-use training. Further research is thus needed to explore how the observed effects of motor learning and intermanual transfer change when motor training is performed over a more extended period of time.

## Resource availability

### Lead contact

Further information and requests for resources should be directed to and will be fulfilled by the lead contact, Kimihiro Nakamura (nakamura-kimihiro@rehab.go.jp).

### Materials availability

This study did not generate new unique reagents.

### Data and code availability

• Behavioral data and functional connectivity data are publicly available at the Open Science Framework (OSF, https://osf.io/vzgjc).
• All R codes used for data analysis are available at the OSF (https://osf.io/vzgjc/).
• Any additional information required to reanalyze the data reported in this paper is available from the lead contact upon request.

## Acknowledgments

This work was supported by the Japan Society for the Promotion of Science (Grants-in-Aid for Scientific Research, 24K23818 to S.T., 20K11176 to K.T., and 23H03262 to K.N.).

## Author contributions

Conceptualization, methodology, and funding acquisition, S.T., K.T. and K.N.; investigation, S.T. and K.T.; writing – original draft, S.T.; writing – review and editing and supervision, K.N.; resources, K.T.

## Declaration of interests

The authors declare no conflicts of interest.

## Star★Methods

### Key resources table

**Table:** 

REAGENT or RESOURCE	SOURCE	IDENTIFIER
**Deposited data**		

Behavioral and functional connectivity data	This paper	https://osf.io/vzgjc/
Functional localizer data	This paper	https://osf.io/vzgjc/

**Software and algorithms**		

PsychoPy	https://www.psychopy.org/	Version 2023.2.3
SPSS for Windows	https://www.ibm.com/products/spss-statistics	Version 29.0.1.1
R for statistical computing	https://www.r-project.org/	Version 4.2.3
MATLAB	https://www.mathworks.com/	Version R2017a
Statistical Parametric Mapping (SPM12)	http://www.fil.ion.ucl.ac.uk/spm/	–
MarsBaR toolbox	https://marsbar-toolbox.github.io/	Version 0.45
CONN toolbox	https://web.conn-toolbox.org/	Version 22a

### Experimental model and study participant details

#### Participants

Thirty-eight healthy adults (21 females, mean (SD) age = 25.97 (6.69) years) participated in the present study. All of them were strongly right-handed, scoring 13–17 points on the Chapman Handedness Inventory.^39^ All of them had normal or corrected-to-normal vision without a previous history of neurological or psychiatric disorders. None of them had any of the general contraindications for MRI scanning, including claustrophobia, pregnancy or medication use. None routinely used hand tools for personal habits or hobbies or had any professional experience with such tools. Two participants were excluded from analyses due to technical errors during data acquisition, leaving 36 participants (21 females; mean age = 26.02 ± 6.80 years) for final analysis. As summarized in Table S2, these participants were matched between the training and control groups for sex, age and years of education. In addition, because resting-state fMRI signals are susceptible to the level of arousal,^40^^,^^41^ we assessed participants’ drowsiness during MRI scanning using verbal inquiry and the Stanford Sleepiness Scale,^42^ which confirmed that none of them fell asleep during MRI data acquisition (Table S2). All participants gave written informed consent prior to the experiment. The protocol of the study was approved by the Research Ethics and Safety Committee of National Rehabilitation Center for Persons with Disabilities (Approval N: 2023016) and conducted in accordance with the Declaration of Helsinki.

### Method details

#### Experimental procedures

The present study was conducted as a single-blind randomized controlled trial, following the experimental procedure illustrated in Figure 2. Participants were randomly assigned to either a tool-use training group or a control training group using a block method with a random number table. Behavioral tasks described below were all performed in a quiet laboratory room.

#### Behavioral paradigm

The behavioral paradigm consisted of two different object manipulation tasks, one for intervention (“tool-use training task”) and the other for pre- and post-intervention behavioral assessments (“assessment task”). During the training task, participants were seated on a 50-cm high standard chair, approximately 15 cm from a 72-cm high desk (Figure 1B). Two round plastic plates (15 cm in diameter and 1 cm in depth) were placed horizontally, 10 cm apart from each other, at approximately 12 cm from the edge of the desk. We used a pair of “reverse-action pliers” purchased from a commercially available source (https://www.ebay.com/itm/196909633233) as a novel tool for intervention and assessment. The special pair of C-ring pliers without holes (maximum opening = 30 mm, jaw length = 45 mm, handle length = 120 mm) is designed to open when squeezed (Figure 1A). Three different types of small wooden blocks, i.e., cubes with a volume of ∼1 cm^3^ (0.7 g), ellipsoids with an axis of ∼1.5 cm × 2 cm (2.8 g) and sticks with an axis of ∼0.5 cm × 3 cm (0.4 g), were also used for the same task. These blocks were all made of the same material with uncoated surfaces.

During intervention, participants in the training and control groups each received 16 cycles of a 30-s trial followed by a 30-s rest. On each trial, a set of nine wooden blocks (three items x three types) were delivered onto one of the two plates placed side by side (Figure 1B). Participants in the training group were instructed to transfer these blocks one-by-one to the opposite plate using the reverse-action pliers in their non-dominant left hand. The nine blocks were placed alternately on the left and right plates across trials, such that participants transferred them from left to right on eight trials and from right to left on eight trials. In contrast, participants in the control group simply held the same pliers in their left hand without moving them and kept their forearms off the table on each trial so that that no motor learning could occur for the novel tool during the same period (“control task”). The experimental setting for training was validated by a previous study which demonstrated reliable behavioral effects of tool-use learning within the short time frame described above.^43^ During the 30-s rest period, all participants were requested to place their left and right hands as well as the tool on the desk and focus on a fixation point displayed on the computer screen. In the training group, behavioral accuracy in tool-use gradually increased over 16 cycles of trial-and-rest during intervention (Figure 3A).

Additionally, it is noteworthy that while intermanual transfer can occur well in both directions between the dominant and non-dominant hand,^44^^,^^45^ we chose to train the non-dominant hand in the present study for the following reasons. First, we had previously validated a similar experimental setting for training the non-dominant left hand,^43^ which can produce reliable behavioral effects of tool-use learning within the short time frame required for the study. Second, by examining neural effects of left-hand training, we could directly examine the callosal access model of intermanual transfer, which posits that motor memory is primarily represented in the left hemisphere regardless of which hand is trained (see Introduction).

A slightly different experimental setting was deliberately used for the pre- and post-intervention behavioral assessment task (Figure 1C), since our main interest was to look at the generalization and transfer of abstract knowledge of tool-use skill. That is, participants were seated on a 42-cm high standard chair, approximately 15 cm from a 60-cm high desk. Unlike in the training and controls tasks described above, two round plastic plates were placed in the front-to-back direction on the desk and connected to a laptop PC via an Arduino Nano microcontroller (https://www.arduino.cc/). Twenty wooden balls of ∼1 cm diameter (0.7 g), all made of the same material as those used for the training task, were used for the assessments. Participants were instructed to transfer these balls one-by-one from the front plate to the back plate using the reverse-action pliers in their left or right hand. By varying functional requirements of tasks between intervention and assessment, we attempted to minimize possible influences from sensory and motor habituation specific to tasks, which allowed us to focus on more abstract representations of tool-use skill beyond task-specific effects of learning. We measured the time required to transfer all of the 20 balls to the destination plate (“transfer time”) by monitoring temporal changes in the weight of plates (sampling rate = 86 Hz, Figure 1D). This transfer time was thus recorded with ∼12 ms precision and used as a behavioral index of learned tool-use skill for each participant.

As illustrated in Figure 2, participants first received the pre-intervention assessment in which they performed the assessment task immediately after rsfMRI scanning (see below). They then performed either the training task or control task with their left hand during intervention. The post-intervention assessment included the same set of the assessment task and rsfMRI scans as the pre-intervention assessment. Importantly, participants performed the assessment task first with their right hand and then with their left hand in the pre-intervention assessment, whereas the order of hands was reversed in the post-intervention assessment (Figure 2).

#### fMRI procedures

Imaging data were acquired using a Magnetom Skyra 3 T scanner with a 64-channel head coil imaging (Siemens Healthcare, Erlangen, Germany). Functional images were acquired using multi-band 2D echo-planar imaging with the following parameters: repetition time = 1230 ms, echo time = 30 ms, flip angle = 68°, field-of-view = 192 × 192 × 159.6 mm, voxel size = 3 × 3 × 3 mm and 40 slices. High-resolution anatomical images were also obtained using a T1-weighted MPRAGE sequence with the following parameters: repetition time = 2300 ms, echo time = 2.98 ms, flip angle = 9°, field-of-view = 248 × 256 × 193.6 mm, voxel size = 1 × 1 × 1.1 mm and 176 slices. All visual cues for behavioral control were generated using PsychoPy (https://www.psychopy.org/). During the pre- and post-intervention assessments (Figure 2), participants first underwent rsfMRI, lasting ∼13 min and yielding 660 functional volumes, in which they passively viewed a fixation point on the screen without engaging in focused thought.

Following the post-intervention assessment, participants underwent functional localization scans designed to identify brain areas associated with tool-use (Figure 2). This block-design task-based fMRI experiment used a simple motor task with a different tool and consisted of 12 task blocks alternating with 12 rest blocks every 28 s. Here, we deliberately used a different motor task from the training and assessment tasks described above, because these tasks were difficult to implement in the MRI environment. For example, the metal reverse-action pliers are incompatible with MRI. Participants would be also unable to see objects and manipulate the tool within the confined space of the MRI scanner. We therefore employed a “chopstick manipulation task” which has been used to isolate neural systems involved in complex finger/hand movements during tool-use.^43^^,^^46^^,^^47^ Participants were given a pair of chopsticks in each hand before the start of the localizer session. During task blocks, they opened and closed the chopsticks in either hand every 2 s according to visual cues displayed on the screen, whereas they held the chopsticks without moving them during rest blocks. The 12 task blocks were alternated between the left and right hands, with six blocks being assigned to each hand.

### Quantification and statistical analysis

All behavioral data were analyzed using SPSS version 29.0.1.1 for Windows. Imaging data were preprocessed and analyzed using SPM12 (http://www.fil.ion.ucl.ac.uk/spm/). Images from each participant were corrected for slice timing and head movements, normalized to the Montreal Neurological Institute template with a 2 × 2 × 2 mm^3^ voxel size and spatially smoothed with an isotropic Gaussian filter (8 mm width at half maximum). Brain regions involved in tool-use were first identified using the functional localizer data described above. For each participant, a weighted-mean image for the tool-use condition (relative to the rest) was computed for each hand by fitting each voxel time-series with the known boxcar function convolved with a canonical hemodynamic response function. Six motion parameters for head motion were included as nuisance regressors. A high-pass filter with a cutoff of 128 s was applied to remove low-frequency drifts.

In group-level analysis, those weighted-mean contrast images from all participants were submitted to one-way within-subject ANOVA to identify brain regions showing greater activation for the left-hand movements relative to the right-hand movements. The resulting statistical map (Figure 4A) was used to identify the local maximum in five regions-of-interest (ROIs) associated with tool-use in each hemisphere by reference to the existing neuroimaging data, i.e., the PMd,^48^^,^^49^^,^^50^ intraparietal sulcus (IPS),^30^^,^^51^ SMA,^9^^,^^49^^,^^52^ basal ganglia (BG)^53^^,^^54^ and cerebellum (CB).^52^^,^^53^ Using the MarsBaR toolbox (https://marsbar-toolbox.github.io/), five 7-mm radius ROIs were then created at x = ±22, y = −8, z = 60 for PMd, x = ±40, y = −40, z = 52 for IPS, x = ±50, y = −28, z = 38 for SMA, x = ±10, y = −6, z = −4 for BG and x = ±18, y = −54, z = −24 for CB in the left and right hemispheres (Figure 4B).

RSFC analysis was performed using the CONN toolbox.^55^ Following the preprocessing pipeline described above, a conventional band-pass filter within a low-frequency window of interest (0.008–0.09 Hz) was applied to the rsfMRI time-series data. A CompCor strategy was implemented to extract noise components from white matter and cerebrospinal fluid using principal component analysis, while keeping intact the strength of intrinsic functional connectivity in the gray matter.^56^ These noise components along with the head motion parameters were included as nuisance covariates.

Since complex hand motor skills are known to primarily rely on the contralateral PMd,^14^^,^^57^ we first searched for brain regions showing changes in RSFC with PMd ROIs after intervention. This was achieved for each of the left and right PMd seeds using a whole-brain 2 × 2 ANOVA with the effect of Period (before vs. after intervention) as a within-participant factor and the effect of Group (training vs. control) as a between-participants factor (voxel-level *p* < 0.001 uncorrected, cluster-level *p* < 0.05 corrected with false discovery rate (FDR) for multiple comparisons). Since this initial analysis revealed no brain region showing significant Period × Group interaction, we restricted the search volume to those ten ROIs described above (FDR-corrected *p* < 0.05, Table S3). However, we again found no brain region showing significant Period × Group interaction in RSFC, either for the left PMd seed or for the right PMd seed.

While tool-use performance uniformly improved after intervention across participants in the training group, this does not necessarily imply that underlying neural connectivity also increased within each participant. Namely, it is possible that connection strength increased in some participants but decreased in others after intervention, because patterns of local functional connectivity might vary among individuals. We therefore used a standard parametric approach^58^^,^^59^^,^^60^^,^^61^ to explore functional connectivity changes which correlated with behavioral measures of tool-use learning for each hand. This is because robust behavioral effects of tool-use learning (Figure 5) may serve as a more fine-grained model for detecting learning-related changes in RSFC than the simple learning × group interaction described above.

Specifically, Pearson’s correlation coefficients in fMRI time-series data were calculated between each PMd seed and all other ROIs for each participant. The resulting correlation coefficients were transformed into normally distributed Z scores using Fisher’s method. For each ROI, learning-related changes in RSFC were then calculated for each PMd seed by subtracting the Z scores for the pre-intervention scans from those for the post-intervention scans. To assess the effects of RSFC on behavior, we computed correlations between these difference scores and intervention-induced reduction in transfer time for each hand for each group and then ran Z-tests to determine whether the resulting RSFC-behavior correlations differed in strength between the two groups (Table 1).

Since this analysis identified three right-hemisphere regions associated with the untrained right-hand performance (i.e., PMd, IPS and BG), we performed an additional analysis to formally compare the RSFC-behavior correlation strengths between these sites. This was achieved using the coranova package for R (https://github.com/gunns2/coranova), which allowed ANOVA-like comparisons for correlation coefficients between groups and conditions.^62^^,^^63^ All other correlation analyses and their between-group comparisons were conducted using SPSS (see above) and corrected for multiple comparisons with FDR.
