# Supplementary material for: The basal ganglia mediate the inter-hemispheric transfer of complex tool-use skill
Source: iScience. 2025 Dec 23;29(2):114523. doi: 10.1016/j.isci.2025.114523 (PMC12818111; doi:10.1016/j.isci.2025.114523)
Supplement: Document S1. Tables S1–S3 [file mmc1.pdf]

iScience, Volume *29*

Supplemental information

The basal ganglia mediate the inter-hemispheric transfer of complex tool-use skill

Sayori Takeda, Kouji Takano, and Kimihiro Nakamura

Table S1. Supplemental analyses for each hand and for each group. A 2 x 2 ANOVA was performed for each hand by treating the effects of Intervention and Group as within- and between-participants factors, respectively (Top). Likewise, a 2 x 2 ANOVA was performed separately for each group with the effects of Hand and Intervention as within-participants factors (Bottom).

	Main effects and interaction	*F*	η^2_p	*p*
**Hand**				
Left	Intervention	54.92	0.618	< 0.001
	Group	0.55	0.016	0.464
	Intervention x Group	12.77	0.273	< 0.001
Right	Intervention	84.38	0.713	< 0.001
	Group	2.74	0.075	0.107
	Intervention x Group	7.48	0.180	< 0.01
**Group**				
Training	Hand	0.33	0.019	0.573
	Intervention	66.90	0.797	< 0.001
	Hand x Intervention	15.89	0.483	< 0.001
Control	Hand	1.61	0.086	0.222
	Intervention	32.75	0.658	< 0.001
	Hand x Intervention	5.76	0.253	0.028

Table S2. Demographic characteristics and sleepiness scores for the training and control groups. Sleep duration for the night before the study was self-reported by each participant. The Stanford Sleepiness Scale was measured immediately after intervention. Sex ratio was compared between the two groups using a chi-square test. All other between-group comparisons were performed using t-tests.

	Training *Mean (SD)*	Control *Mean (SD)*	*p* value
Age (years)	26.4 (6.3)	25.7 (7.7)	0.759
Sex (M/F)	8 M / 10 F	7 M / 11 F	0.735
Chapman’s handedness scale	13.2 (0.5)	13.2 (0.5)	1.000
Education (years)	17.8 (2.2)	17.2 (1.4)	0.284
Sleep duration (hours)	6.8 (0.6)	7.0 (1.7)	0.649
Stanford Sleepiness Scale	3.6 (1.1)	3.9 (1.1)	0.286

Table S3 Changes in RSFC with left and right PMd seeds before and after intervention. Mean connection strengths (SD) are presented for each ROI with respect to the left and right PMd seeds. All *p* values are FDR-corrected for multiple comparisons.

Seed	ROI	Group	RSFC (Z)		Group x Intervention interaction		
			Before	*After*	*F*	η^2_p	*p*
			Mean (SD)	*Mean (SD)*			
L PMd	R PMd	Training	0.61 (0.23)	0.63 (0.20)	1.42	0.040	0.726
		Control	0.56 (0.16)	0.66 (0.19)			
	L SMA	Training	0.46 (0.19)	0.40 (0.19)	1.63	0.046	0.940
		Control	0.33 (0.21)	0.33 (0.21)			
	R SMA	Training	0.35 (0.18)	0.30 (0.22)	0.15	0.004	0.787
		Control	0.33 (0.20)	0.30 (0.22)			
	L IPS	Training	0.39 (0.24)	0.40 (0.29)	0.05	0.001	0.832
		Control	0.22 (0.28)	0.22 (0.30)			
	R IPS	Training	0.24 (0.19)	0.15 (0.15)	1.38	0.039	0.555
		Control	0.08 (0.19)	0.05 (0.26)			
	L BG	Training	-0.03 (0.13)	0.02 (0.13)	3.48	0.093	0.639
		Control	-0.00 (0.10)	-0.04 (0.09)			
	R BG	Training	-0.01 (0.17)	-0.02 (0.14)	0.33	0.011	0.731
		Control	-0.01 (0.80)	-0.05 (0.09)			
	L CB	Training	0.07 (0.10)	0.09 (0.12)	0.55	0.016	0.693
		Control	0.06 (0.12)	0.10 (0.18)			
	R CB	Training	0.07 (0.11)	0.11 (0.90)	0.67	0.020	0.748
		Control	0.12 (0.14)	0.12 (0.16)			
R PMd	L PMd	Training	0.61 (0.23)	0.63 (0.20)	1.42	0.040	0.726
		Control	0.56 (0.16)	0.66 (0.19)			
	L SMA	Training	0.37 (0.16)	0.32 (0.19)	3.06	0.083	0.801
		Control	0.33 (0.16)	0.37 (0.16)			
	R SMA	Training	0.51 (0.21)	0.47 (0.18)	0.04	0.001	0.929
		Control	0.55 (0.24)	0.50 (0.20)			
	L IPS	Training	0.24 (0.22)	0.23 (0.19)	0.23	0.060	1.132
		Control	0.12 (0.19)	0.14 (0.26)			
	R IPS	Training	0.14 (0.18)	0.06 (0.16)	2.18	0.002	0.670
		Control	0.08 (0.14)	0.09 (0.20)			
	L BG	Training	-0.02 (0.15)	-0.00 (0.13)	0.02	0.0004	0.902
		Control	-0.02 (0.10)	-0.01 (0.10)			
	R BG	Training	-0.05 (0.18)	-0.08 (0.13)	0.15	0.004	1.042
		Control	-0.02 (0.09)	-0.06 (0.10)			
	L CB	Training	0.13 (0.17)	0.16 (0.11)	0.90	0.026	0.785
		Control	0.13 (0.15)	0.11 (0.16)			
	R CB	Training	0.06 (0.11)	0.09 (0.10)	0.07	0.002	1.023
		Control	0.06 (0.14)	0.08 (0.12)			
